# Mechanical Compression Versus Vascular Closure Devices for Femoral Artery Haemostasis After Peripheral Endovascular Procedures: A Randomised Controlled Trial

**DOI:** 10.3390/jcm15114197

**Published:** 2026-05-29

**Authors:** Irina Shevchenko, Bernardette Jingfei Lee, Davina Daudu, James Dodd, Jackie Wong, Olufemi Ayoadeleke Oshin, Fernando Picazo-Pineda, Mahmoud Al-Najjar, Tanya Michelle Rhine, Carolina Bravo Ceballos, Bibombe Patrice Mwipatayi

**Affiliations:** 1 Cabrini Hospital, 183 Wattletree Road, Malvern, VIC 3144, Australia; 2 Department of Vascular and Endovascular Surgery, Sir Charles Gairdner Hospital, Perth, WA 6009, Australiajames.dodd@health.wa.gov.au (J.D.);; 3 Department of Vascular and Endovascular Surgery, Royal Perth Hospital, Perth, WA 6000, Australiaolufemi.oshin@health.wa.gov.au (O.A.O.);; 4 Australian Vascular, Perth, WA 6000, Australia; 5 Rural Clinical School of Western Australia, University of Western Australia, Perth, WA 6009, Australia; 6 School of Surgery, RPH Research Foundation Building 56, Murray Street, Perth, WA 6000, Australia

**Keywords:** femoral artery haemostasis, vascular closure devices, FemoStop II Gold, peripheral endovascular procedures, access-site complications, randomised controlled trial

## Abstract

**Background:** Femoral arteriotomy closure after peripheral angiography and intervention is commonly achieved using vascular closure devices (VCDs) or compression-based strategies; however, comparative randomised data in contemporary peripheral endovascular practice remain limited. **Methods:** In this prospective randomised trial, adults undergoing femoral-access diagnostic angiography or peripheral endovascular intervention were assigned in a 1:1 ratio to haemostasis with the FemoStop™ II Gold pneumatic compression system or a contemporary VCD strategy. The primary endpoint was a composite of major or minor groin-site complications immediately after sheath removal. Secondary endpoints included composite complications at recovery, discharge, and 30 days, with separate analyses of major and minor complications. Patient-reported pain was assessed using the Verbal Numerical Rating Scale (VNRS). Efficacy and safety analyses were performed according to the intention-to-treat and as-treated principles, respectively. Risk ratios were estimated using modified Poisson regression with robust variance, with prespecified adjustment for sex, systolic blood pressure before sheath removal, and sheath size. **Results:** A total of 130 participants underwent randomisation, including 66 assigned to FemoStop™ II Gold and 64 assigned to VCDs. The primary composite endpoint occurred in 23/66 participants (34.9%) in the FemoStop™ II Gold group and 16/64 (25.0%) in the VCD group (absolute difference, 9.9 percentage points; 95% confidence interval [CI], −6.1 to 25.7; *p* = 0.25), with the numerical difference driven predominantly by minor-only events (28.8% versus 15.6%; *p* = 0.09). At 30 days, the composite endpoint occurred in 17/66 participants (25.8%) and 12/64 participants (18.8%), respectively (absolute difference, 7.0 percentage points; 95% CI, −13.3 to 26.4; *p* = 0.40). Serious access-site events remained infrequent both immediately post-procedure (6.1% versus 9.4%; *p* = 0.53) and at 30 days (6.1% versus 4.7%; *p* = 0.72). The adjusted risk ratios were 1.28 (95% CI, 0.74 to 2.21) for the primary composite endpoint and 1.23 (95% CI, 0.63 to 2.40) for the 30-day composite endpoint. Ordinal VNRS pain distributions did not differ significantly at any timepoint, although “any pain” immediately post-procedure was less frequent with FemoStop™ II Gold (22.7% versus 40.6%; unadjusted risk ratio, 0.56; 95% CI, 0.33 to 0.93); this association was attenuated after adjustment (adjusted risk ratio, 0.63; 95% CI, 0.38 to 1.03). Prespecified interaction testing suggested that the effect of treatment on composite complications varied according to sheath size both immediately post-procedure and at 30 days (*p* < 0.001 for both interactions). **Conclusions:** In patients undergoing femoral-access diagnostic angiography or peripheral endovascular intervention, haemostasis with FemoStop™ II Gold resulted in 30-day groin-site complication rates that did not differ significantly from those observed with contemporary VCD strategies. Serious access-site events remained infrequent in both groups, and the apparent early reduction in patient-reported pain with FemoStop™ II Gold was not definitive after adjustment. Larger, adequately powered multicentre studies are warranted to clarify sheath size-dependent effects and uncommon clinically consequential vascular events.

## 1. Introduction

Peripheral arterial disease (PAD) affects more than 200 million individuals worldwide and is predominantly caused by progressive atherosclerosis resulting in stenosis or occlusion of peripheral arteries, particularly within the lower extremities [1,2,3,4]. In Australia, cardiovascular disease, including PAD, accounted for approximately one-quarter of all deaths in 2019 [5]. Beyond limb-related morbidity, lower-extremity arterial disease contributes substantially to recurrent hospitalisation, reduced mobility, impaired quality of life, increased healthcare utilisation, and elevated long-term cardiovascular mortality.

The diagnosis of PAD is commonly established via angiography. As part of this process, contrast material is administered within the arterial system to define the anatomical extent and severity of disease [6]. Over the past two decades, endovascular revascularisation with balloon angioplasty has become the principal therapeutic strategy for symptomatic PAD, providing a minimally invasive alternative to open surgical bypass [7]. In selected lesions, adjunctive stent implantation may be required to maintain vessel patency and optimise procedural durability [7]. Regardless of the revascularisation strategy employed, common femoral arterial access remains the dominant procedural approach, and subsequent sheath removal necessitates reliable haemostasis at the arteriotomy site [8].

Arterial closure after femoral access may be achieved using manual compression, mechanical compression systems, or vascular closure devices (VCDs) [9,10]. Each approach carries inherent trade-offs relating to patient comfort, time to haemostasis, early ambulation, workflow efficiency, healthcare resource utilisation, and the risk of access-site complications [10,11]. Although severe complications such as pseudoaneurysm formation, retroperitoneal haemorrhage, arterial thrombosis, infection, or access-site occlusion are relatively uncommon, they may result in the need for blood transfusion, prolonged hospitalisation, emergency intervention, or surgical repair [12,13]. Minor complications are considerably more frequent and, although often self-limited, contribute substantially to patient discomfort, delayed mobilisation, intensified nursing observation, and increased post-procedural resource utilisation [12,14].

Contemporary cardiovascular guidance recognises the importance of optimal femoral access management in reducing procedural morbidity, facilitating early mobilisation, and minimising bleeding complications. The American Heart Association’s scientific statement on arteriotomy closure devices emphasises careful access-site management, appropriate selection of haemostasis strategy, and consideration of anatomical characteristics, sheath calibre, anticoagulation status, and operator experience [9], principles which have become increasingly relevant within modern endovascular care, where progressively older and more comorbid patients undergo increasingly complex procedures requiring larger sheath sizes, intensified antithrombotic therapy, prolonged procedural duration, and advanced revascularisation techniques.

Although manual compression remains widely practised because of its simplicity and universal availability, this technique is labour-intensive, may prolong immobilisation, and is frequently associated with patient discomfort and increased nursing workload [10,14]. The FemoStop™ II Gold compression system was developed as a hands-free mechanical alternative designed to provide controlled external pressure and reduce reliance on prolonged manual compression [14]. VCDs, in contrast, facilitate earlier ambulation and shorter times to haemostasis, but may also be associated with device-specific complications, including bleeding, infection, pseudoaneurysm formation, arterial occlusion, and device failure [9,10,11].

Despite widespread adoption of these haemostatic strategies, robust comparative evidence evaluating mechanical compression systems within current peripheral intervention practice remains limited. Published studies have focused predominantly on VCDs and have frequently relied on retrospective or observational designs with heterogeneous definitions of access-site complications, inconsistent follow-up intervals, and variable reporting of clinically relevant outcomes [10,11,12,15]. Furthermore, patient-reported outcomes such as procedural pain, post-procedural comfort, and early mobilisation, although clinically important, have been inconsistently evaluated [10,11].

We, therefore, conducted a prospective randomised controlled trial to evaluate whether a structured mechanical compression strategy using the FemoStop™ II Gold system could achieve clinically acceptable femoral haemostasis outcomes compared with contemporary vascular closure device strategies in patients undergoing peripheral endovascular intervention. We hypothesised that FemoStop™ II Gold would provide similar effectiveness in reducing groin puncture-site complications, while potentially differing from vascular closure devices with respect to patient-reported discomfort, post-procedural care requirements, and procedural resource utilisation. By evaluating femoral-access haemostasis strategies within a controlled clinical framework, this study aimed to provide clinically relevant evidence to support haemostasis strategy selection, inform post-procedural monitoring pathways, and optimise peri-procedural care in contemporary peripheral endovascular practice.

## 2. Methods

### 2.1. Trial Design and Oversight

We conducted a prospective, open-label, parallel-group randomised controlled trial comparing the FemoStop™ II Gold femoral compression system (Abbott Cardiovascular, Plymouth, MN, USA) with vascular closure devices for haemostasis following common femoral arterial access in patients undergoing endovascular procedures for PAD. Participants were assigned in a 1:1 ratio.

The study protocol and prespecified statistical analysis plan were approved by the Human Research Ethics Committees of Royal Perth Hospital, Hollywood Private Hospital, and La Trobe University (approval code RGS0000004119; approval date, 2 June 2020). The trial was prospectively registered with the Australian New Zealand Clinical Trial Registry (ACTRN12620000784910) on 3 August 2020. The study was conducted in accordance with the Declaration of Helsinki and guidelines of Good Clinical Practice, and written informed consent was obtained from all participants prior to enrolment.

The investigators designed the study, collected and analysed the data, and prepared the manuscript. The authors attest to the completeness and accuracy of the data and the trial’s adherence to the protocol. This study is reported in accordance with the CONSORT 2025 statement, and the completed checklist is provided in Supplementary Table S1.

### 2.2. Patients

Trial procedures were performed at Royal Perth Hospital and Hollywood Private Hospital, Perth, Western Australia. Interventions were delivered by appropriately trained vascular surgeons and endovascular specialists according to institutional standards of care.

Adults aged 18 years or older scheduled to undergo elective or emergency endovascular procedures requiring common femoral arterial access were eligible for inclusion. All participants were required to provide written informed consent and adhere to the study follow-up schedule.

Exclusion criteria included inability to comply with follow-up, pregnancy, anticipated need for sheath upsizing beyond 7 French, thrombolytic therapy before the procedure, ongoing therapeutic heparin infusion, haemodynamic instability defined as systolic blood pressure exceeding 200 mmHg, prior intervention at the same femoral access site within the preceding month, and active dermatological conditions affecting the puncture site.

### 2.3. Randomisation and Study Procedures

Following completion of the index endovascular procedure and confirmation of ongoing eligibility, participants were randomly assigned to haemostasis with either the FemoStop™ II Gold compression system or a vascular closure device strategy. The allocation sequence was computer-generated with equal allocation and was prepared by an independent hospital research support unit not involved in recruitment, procedural management, data collection, or statistical analysis.

Allocation concealment was achieved using consecutively numbered opaque sealed envelopes, which were opened only after patients were enrolled and immediately before sheath removal. Because of the procedural nature of the interventions, blinding of participants, proceduralists, and treating staff was not feasible.

In the FemoStop™ II Gold group, haemostasis was achieved using pneumatic compression according to the manufacturer’s recommended protocol with a gradual reduction in dome pressure under monitored conditions. In the VCD group, haemostasis was achieved using an approved closure device selected according to operator preference and institutional practice.

**Figure 1:**
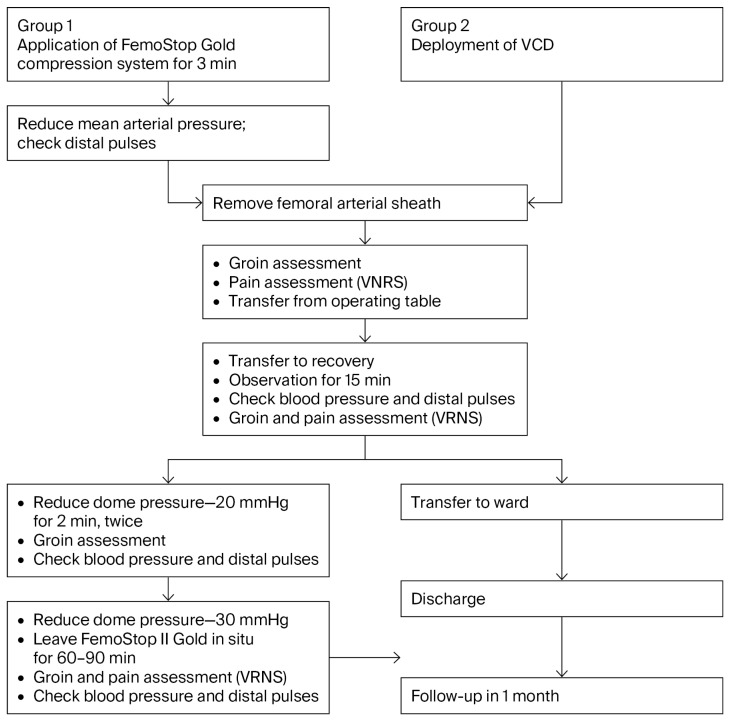
The treatment flowchart shown are the standardised haemostasis and post-procedural monitoring pathways for the FemoStop™ II Gold compression strategy and the vascular closure device strategy, including sheath removal, structured groin and haemodynamic assessment, serial reassessment during recovery, ward transfer, discharge, and 1-month follow-up.

Participants subsequently entered a structured post-procedural monitoring pathway designed to identify early access-site complications and haemodynamic instability. Immediately after sheath removal, the puncture site was assessed for bleeding, haematoma formation, and distal limb perfusion. Blood pressure was measured, and a distal pulse examination was conducted. Serial reassessments were performed in the recovery area at predefined intervals before ward transfer and discharge. All participants underwent formal clinical review at 30 days to identify delayed vascular complications, including pseudoaneurysm formation and retroperitoneal haemorrhage. The procedural haemostasis algorithm is illustrated in Figure 1. Primary study data were systematically extracted from medical records and entered into a secure, password-protected REDCap database. All data underwent a rigorous de-identification process prior to analysis to ensure the utmost confidentiality.

### 2.4. Endpoints

The primary endpoint was the composite incidence of groin-site vascular complications, major or minor, immediately after arterial sheath removal following femoral arteriotomy.

Minor complications included oozing, ecchymosis, small haematoma, large haematoma, and bleeding that did not require invasive intervention. Major complications included pseudoaneurysm formation and retroperitoneal haemorrhage confirmed clinically and/or radiologically [12,13].

Secondary endpoints included composite groin-site complications at recovery, discharge, or 30 days, together with separate analyses of major and minor complications at each timepoint. Individual complication factors were also analysed.

Patient-reported pain was assessed at all in-hospital timepoints using the Verbal Numerical Rating Scale (VNRS; range 0–10). Pain severity was categorised as no pain (0), mild pain (1–3), moderate pain (4–6), and severe pain (7–10). A prespecified dichotomous outcome of any pain (VNRS ≥ 1) was additionally analysed.

Prespecified exploratory analyses evaluated the modification of potential effects according to sheath size and timepoint.

### 2.5. Statistical Analysis

All efficacy analyses were performed according to the intention-to-treat principle, whereby participants were analysed according to their randomly assigned group, irrespective of the haemostasis strategy ultimately received [16,17]. Safety analyses were performed in the as-treated population.

Continuous variables are presented as means ± standard deviations, or medians with interquartile ranges where appropriate. Between-group comparisons were performed using Student’s *t*-test or the Wilcoxon rank-sum test. Categorical variables are presented as counts and percentages and were compared using the chi-square test or Fisher’s exact test.

Sample size determination was based on the anticipated between-group difference in the primary composite outcome. Assuming an event rate of approximately 40% in the VCD group and an absolute risk reduction of 18 percentage points, a total sample of 124 participants would provide 80% power to detect this difference at a two-sided alpha level of 0.05 [18]. To account for attrition and incomplete follow-up, the enrolment target was increased to 130 participants.

Relative risks were estimated using modified Poisson regression with robust variance estimation, as described by Zou [19]. Adjusted analyses incorporated prespecified covariates, including sex, systolic blood pressure before sheath removal, and sheath size. Ordinal VNRS pain outcomes were analysed using proportional-odds logistic regression [20], and ordinal effect sizes were quantified using Cramér’s V [21].

Prespecified interaction analyses evaluated treatment-by-sheath-size and treatment-by-time interactions using multiplicative interaction terms within regression models. Given the limited statistical power for interaction testing, these analyses were considered exploratory.

All tests were two-sided, and *p*-values < 0.05 were considered statistically significant. Statistical analyses were performed using Stata version 18 (StataCorp).

## 3. Results

**Figure 2:**
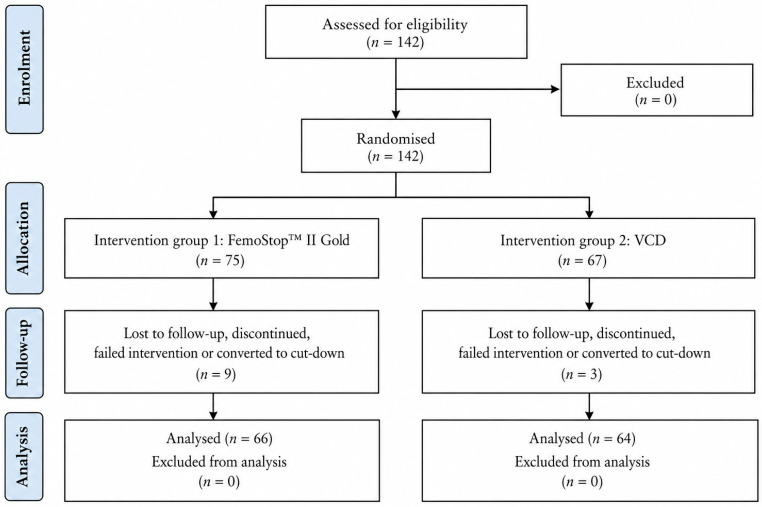
Participant flow throughout the study, from screening, to randomisation, treatment allocation, follow-up, and analysis phases. Of the 142 participants assessed and randomised, 75 and 67 were assigned to the FemoStop™ II Gold and the vascular closure device (VCD) groups, respectively. During follow-up, 3 participants in the FemoStop group were lost to follow-up, while 6 discontinued the assigned intervention. In the VCD group, no participants were lost to follow-up; however, 3 discontinued the intervention due to device failure, requiring conversion. A total of 130 participants were included in the primary analyses. Recruitment took place between December 2020 and November 2021, and the final 30-day follow-up was completed in December 2021.

A total of 142 participants underwent randomisation, including 75 assigned to the FemoStop™ II Gold group and 67 assigned to the vascular closure device (VCD) group (Figure 2). All participants initially underwent the allocated haemostasis strategy. Following randomisation, 12 participants were excluded from the final analysed cohort because of loss to follow-up, procedural conversion, or discontinuation of the allocated intervention. In the FemoStop™ II Gold group, three participants were lost to follow-up and six discontinued the allocated intervention because of conversion to cut-down or manual compression. In the VCD group, three participants discontinued the allocated intervention because of device failure requiring conversion. The final analysed cohort, therefore, comprised 130 participants, including 66 assigned to FemoStop™ II Gold and 64 assigned to VCDs; this flow of participants, including post-randomisation exclusions and reasons for discontinuation, is shown in Figure 2.

Baseline clinical characteristics were well balanced between groups (Table 1). The mean age of the cohort was 68.5 ± 10.7 years, and 72% of participants were male. Cardiovascular comorbidities were substantial, including hypertension (75%), hyperlipidaemia (54%), type 2 diabetes mellitus (66%), coronary artery disease (49%), prior myocardial infarction (25%), atrial fibrillation/flutter (22%), and previous peripheral intervention (72%). Antithrombotic therapy reflected routine contemporary management, with 51% receiving single antiplatelet therapy, 32% receiving dual antiplatelet therapy, and 15% receiving direct oral anticoagulants.

The study cohort, therefore, represented a clinically complex PAD population with extensive systemic vascular disease and substantial cardiovascular comorbidity. Although heart failure status was not systematically classified according to New York Heart Association functional class, the overall cardiovascular profile of the cohort reflected elevated procedural and bleeding risk.

Procedural characteristics were similarly distributed between groups (Table 2). Most procedures were interventional, rather than diagnostic (78.5%). The procedural mix included plain balloon angioplasty, drug-coated balloon angioplasty, bare-metal stenting, covered stenting, and atherectomy combined with drug-coated balloon angioplasty. Many procedures were performed using sheath sizes of 5–6 Fr.

The primary composite endpoint occurred in 23 out of 66 participants (34.9%) in the FemoStop™ II Gold group and 16 out of 64 participants (25.0%) in the VCD group (absolute difference, 9.9 percentage points; 95% confidence interval [CI], −6.1 to 25.7; *p* = 0.25) (Table 3). When analysed hierarchically, serious access-site events occurred in 6.1% and 9.4% of participants, respectively, whereas minor-only complications occurred in 28.8% and 15.6%, respectively. The numerical difference in the composite outcome was, therefore, predominantly driven by minor vascular findings, rather than severe complications.

At recovery, composite complication frequencies remained numerically higher in the FemoStop™ II Gold group than in the VCD group; however, observed between-group differences did not reach statistical significance. By discharge, complication rates had declined substantially in both groups, and serious vascular outcomes remained infrequent. At 30 days, the secondary composite outcome occurred in 25.8% and 18.8% of participants, respectively, without statistically significant between-group differences. Adjusted analyses were concordant with the unadjusted findings (Table 4). The adjusted risk ratio for the primary composite outcome was 1.28 (95% CI, 0.74–2.21), while the adjusted risk ratio for the 30-day composite outcome was 1.23 (95% CI, 0.63–2.40). Confidence intervals remained relatively broad because of the low absolute frequency of serious vascular events. 

Participant-reported pain distributions are shown in Table 5. Ordinal VNRS pain distributions did not differ significantly between groups at any timepoint. Immediately after the procedure, however, fewer participants reported experiences of pain in the FemoStop™ II Gold group compared with the VCD group (22.7% versus 40.6%); this apparent association, attenuated after adjustment for prespecified covariates, was not sustained during recovery or discharge.

Over the 30 days, clinically consequential vascular events remained infrequent in both groups. Open surgical repair was required in one participant from each treatment group, whereas ultrasound-guided thrombin injections occurred only within the VCD group. Overall, there was no statistically significant difference in severe vascular outcomes during follow-up.

Exploratory analyses identified statistically significant treatment-by-sheath-size interaction effects for composite groin-site complications immediately after the procedure and at 30-day follow-up (Supplementary Table S5), whereas no evidence of treatment-by-time interaction was observed. Given the limited sample size and restricted power for subgroup and interaction testing, these findings should be regarded as exploratory and hypothesis-generating, rather than confirmatory. Within the VCD arm, closure devices included Angio-Seal™, MynxGrip™, FemoSeal™, and Perclose ProGlide™. Post-procedural complication frequencies were broadly similar across these platforms, with overlapping exact confidence intervals and no statistically significant difference between device subtypes on exploratory Fisher’s exact testing (*p* = 0.94; Supplementary Table S6).

Overall, across primary, secondary, and adjusted analyses, no statistically significant differences were observed between FemoStop™ II Gold and VCD strategies in composite groin-site complications during the follow-up period. Although early post-procedural pain was numerically lower after using FemoStop™ II Gold, this association attenuated after adjustment and was not sustained over time.

## 4. Discussion

In this prospective randomised controlled trial involving patients undergoing femoral-access diagnostic angiography and peripheral endovascular intervention, haemostasis with the FemoStop™ II Gold pneumatic compression system resulted in groin-site complication rates that were clinically and statistically comparable with those observed with contemporary vascular closure device (VCD) strategies during hospitalisation and at 30-day follow-up. Although the primary composite endpoint occurred numerically more frequently in the FemoStop™ II Gold group immediately after sheath removal, the observed difference was modest, statistically non-significant, and driven predominantly by minor vascular findings, rather than severe access-site complications. Importantly, serious vascular outcomes remained infrequent throughout all prespecified assessment periods, and adjusted analyses demonstrated no evidence of excess vascular harm associated with the compression-based strategy.

Contemporary cardiovascular guidance and the broader evidence base increasingly emphasise that femoral arteriotomy closure should not be regarded merely as a technical endpoint, but rather as an important determinant of procedural safety, early mobilisation, post-procedural recovery, and healthcare resource utilisation. The American Heart Association scientific statement on arteriotomy closure devices highlights the importance of careful access-site management, appropriate haemostasis strategy selection, and individualisation according to vascular anatomy, sheath calibre, anticoagulation exposure, and operator experience [9]; these considerations are particularly relevant in current peripheral arterial intervention, where patients frequently present with advanced systemic atherosclerosis, calcified access vessels, diabetes mellitus, chronic kidney disease, and exposure to intensive antiplatelet or anticoagulant therapy. Supporting systematic reviews and meta-analyses similarly suggest that although VCDs may shorten time to haemostasis and ambulation, differences in major access-site outcomes relative to compression-based strategies are often modest and context-dependent [10,11].

The principal findings of the present study are broadly concordant with the wider randomised and observational literature evaluating femoral haemostasis strategies. In the ISAR-CLOSURE trial, closure devices demonstrated low overall rates of serious access-site events compared with manual compression following coronary angiography, although between-strategy differences remained limited [22]. Likewise, the CLOSE-UP and CLOSE-UP III studies evaluating FemoSeal™ and MynxGrip demonstrated improvements in procedural efficiency and time to haemostasis without substantial reductions in clinically significant vascular complications [23,24]. Earlier studies evaluating suture-mediated and collagen-based closure systems similarly reported low rates of severe vascular complications when contemporary techniques and structured surveillance pathways were employed [25,26]. Meta-analyses comparing VCDs with manual compression have also shown that although closure devices may facilitate earlier mobilisation and reduce nursing workload, differences in major bleeding, pseudoaneurysm formation, and surgical repair are generally small and inconsistent across procedural settings [10,11].

The statistical findings of the present study support a similar interpretation. Although the unadjusted primary composite complication rate was numerically higher in the FemoStop™ II Gold group, confidence intervals crossed the line of no effect and remained compatible with modest benefit and modest harm. In adjusted modified Poisson regression analyses, the risk ratios for the primary composite endpoint and the composite outcome similarly crossed unity, indicating no statistically significant difference between haemostasis strategies after adjustment for clinically relevant covariates, including sex, systolic blood pressure before sheath removal, and sheath size. The relatively broad confidence intervals observed across several analyses likely reflect the low absolute frequency of serious vascular events, a persistent methodological challenge in femoral-access trials.

The overall complication profile observed in this cohort likely reflects the systematic prospective capture of minor vascular findings within a medically complex PAD population characterised by substantial cardiometabolic burden. Participants exhibited high rates of diabetes mellitus, hypertension, dyslipidaemia, smoking exposure, coronary artery disease, atrial fibrillation or flutter, previous peripheral intervention, and chronic kidney disease-related comorbidities, all of which are recognised contributors to impaired arterial integrity, endothelial dysfunction, vascular calcification, and increased susceptibility to bleeding or haematoma formation following femoral puncture [2,3,4,12,13,15]. Although heart failure status was not systematically classified according to New York Heart Association functional class, the overall cardiovascular profile of the cohort nevertheless supports the interpretation that this represented a clinically complex and medically comorbid population frequently encountered in modern endovascular care.

Procedural complexity likely contributed further to the observed vascular profile. Moderate-to-large sheath sizes were frequently required, and increasing sheath calibre proportionally enlarges arteriotomy diameter while escalating local haemostatic demand [12,15]. In addition, widespread use of antiplatelet and anticoagulant therapy likely amplified vulnerability to access-site bleeding complications. Nevertheless, most complications observed during the study were minor, clinically manageable, and potentially modifiable through optimisation of procedural technique and structured post-procedural surveillance pathways.

The importance of meticulous access-site technique has been further reinforced by studies evaluating ultrasound-guided femoral cannulation. The FAUST trial demonstrated that real-time ultrasound-guided femoral access significantly improves first-pass success and reduces vascular complications compared with fluoroscopic or anatomical landmark guidance alone [27]. Subsequent systematic reviews and meta-analyses similarly confirmed reductions in inadvertent venepuncture, haematoma formation, and access-site bleeding with ultrasound-guided arterial cannulation [26]; these findings support the concept that optimisation of procedural access technique may be at least as important as the choice of haemostasis strategy itself in reducing vascular morbidity following peripheral intervention.

Contemporary evidence additionally supports meticulous puncture-site localisation within the common femoral artery, careful sheath size selection, haemodynamic optimisation during sheath removal, and operator familiarity with the selected haemostasis strategy as important measures capable of reducing access-site complications [8,9,10,11,12]. Within this context, the absence of an apparent increase in severe vascular outcomes associated with FemoStop™ II Gold is reassuring. Although the compression-based strategy demonstrated a numerical increase in certain minor complications, clinically significant outcomes, including pseudoaneurysm requiring intervention, surgical repair, retroperitoneal haemorrhage, and major bleeding remained infrequent in both treatment groups.

Several secondary findings warrant consideration. Exploratory analyses demonstrated statistically significant treatment-by-sheath-size interaction effects for composite complications immediately after the procedure and at 30-day follow-up. Although exploratory, these findings are biologically plausible because increasing sheath calibre proportionally enlarges the diameter of the arteriotomy while increasing local arterial wall stress and procedural haemostasis requirements [12,15]. Prior observational studies have similarly identified larger sheath size as an independent predictor of access-site complications following peripheral vascular intervention [12,15]. However, because the present trial was not specifically powered for subgroup interaction analyses, these findings should be interpreted cautiously and regarded as hypothesis-generating, rather than definitive. Larger multicentre studies are therefore required to clarify sheath size-dependent effects and uncommon clinically consequential access-site events.

The observed low frequency of severe vascular outcomes in both treatment groups likely reflects continuing improvements in modern endovascular care, including advances in procedural imaging, antithrombotic management, device technology, and structured peri-procedural monitoring pathways. Increasing procedural experience, wider adoption of ultrasound-guided access, and greater awareness of bleeding avoidance strategies have collectively contributed to progressive reductions in vascular morbidity across peripheral and cardiovascular intervention [27,28,29,30].

Patient-reported pain outcomes also merit consideration. Although global ordinal pain distributions did not differ significantly between treatment groups, exploratory analyses suggested a modest reduction in immediate post-procedural discomfort with FemoStop™ II Gold. However, this apparent association attenuated after adjustment for clinically relevant covariates and was not sustained during recovery or at discharge, suggesting that durable differences in patient-centred comfort outcomes are likely to be modest and multifactorial.

Several limitations should be acknowledged. First, although this was a prospective randomised trial, the study was powered for a composite vascular outcome that included relatively frequent minor complications, rather than for uncommon but clinically important severe access-site events. Consequently, confidence intervals surrounding estimates for major complications remained relatively wide. Importantly, the trial was not designed or powered as a formal non-inferiority study; therefore, the absence of statistically significant between-group differences should not be interpreted as definitive equivalence between haemostasis strategies. Second, the open-label design may have influenced ascertainment of visually graded minor complications or subjective pain reporting, although structured assessment pathways were used consistently. Third, the VCD arm comprised a heterogeneous mixture of device technologies with differing mechanisms of action and deployment characteristics, reflecting routine real-world practice, rather than a single-device comparison. Exploratory device-level analyses did not demonstrate a statistically significant difference in post-procedural complication frequency across VCD subtypes; however, these findings should be interpreted cautiously because subgroup sample sizes were small and the trial was not powered for device-specific comparisons. Finally, although haemodynamically unstable patients were excluded, the study population remained clinically complex, with substantial cardiovascular comorbidity, reflecting the medically challenging cohorts commonly encountered in peripheral arterial intervention.

From a broader clinical and health system perspective, the findings of this trial may have important practical implications. In an era increasingly focused on value-based care, procedural efficiency, and device-related healthcare expenditure, the absence of clear differences in serious vascular outcomes suggests that selection of haemostasis strategy may reasonably incorporate considerations of operator familiarity, workflow efficiency, institutional preference, device availability, and economic cost without materially compromising short-term vascular safety. Future adequately powered multicentre trials incorporating formal economic analyses, ultrasound-guided access protocols, stratification according to sheath calibre and anticoagulation intensity, and longer-term vascular follow-up will be important to further define the optimal haemostasis strategy for modern peripheral arterial intervention.

## 5. Conclusions

Among patients undergoing femoral-access diagnostic angiography or peripheral endovascular intervention, haemostasis with FemoStop™ II Gold resulted in 30-day groin-site complication rates that were not significantly different from those observed with contemporary vascular closure device strategies. Serious access-site events remained infrequent in both groups, and the observed between-group differences were driven predominantly by minor vascular findings. The reported findings suggest that FemoStop™ II Gold may represent a clinically reasonable device-sparing haemostasis strategy in selected patients undergoing peripheral arterial intervention. Larger, adequately powered multicentre studies are warranted to clarify sheath size-dependent effects and uncommon clinically consequential vascular events.

## Figures and Tables

**Table 1:** Baseline characteristics of the study population.

Characteristic	VCD (*n* = 64)	FemoStop™ II Gold (*n* = 66)	Total (*n* = 130)
Demographics			
Age, years	68.6 ± 10.7	68.3 ± 10.7	68.5 ± 10.7
Male sex, *n* (%)	39 (61%)	54 (83%)	93 (72%)
Body mass index, kg/m^2^	29.3 ± 5.3	28.8 ± 4.9	29.0 ± 5.1
Cardiovascular Risk Factors, History, and Comorbidities			
Hypertension, *n* (%)	48 (75%)	50 (76%)	98 (75%)
Hyperlipidaemia, *n* (%)	37 (58%)	33 (50%)	70 (54%)
Type 1 diabetes, *n* (%)	1 (2%)	3 (5%)	4 (3%)
Type 2 diabetes, *n* (%)	40 (63%)	46 (70%)	86 (66%)
Current smoker, *n* (%)	20 (31%)	21 (32%)	41 (32%)
History of angina, *n* (%)	4 (6%)	5 (8%)	9 (7%)
Prior myocardial infarction, *n* (%)	14 (22%)	18 (27%)	32 (25%)
Coronary artery disease, *n* (%)	31 (48%)	33 (50%)	64 (49%)
History of CABG, *n* (%)	6 (9%)	15 (23%)	21 (16%)
Carotid artery disease, *n* (%)	6 (9%)	4 (6%)	10 (8%)
Previous peripheral intervention, *n* (%)	44 (69%)	50 (76%)	94 (72%)
Atrial fibrillation/flutter, *n* (%)	13 (20%)	15 (23%)	28 (22%)
Dialysis-dependent, *n* (%)	1 (2%)	3 (5%)	4 (3%)
COPD, *n* (%)	15 (23%)	16 (24%)	31 (24%)
History of malignancy, *n* (%)	9 (14%)	9 (14%)	18 (14%)
Lipid Profile and Biomarkers			
Total cholesterol, mmol/L	3.70 ± 1.11	3.57 ± 1.17	3.63 ± 1.14
LDL cholesterol, mmol/L	1.89 ± 0.78	1.87 ± 0.98	1.88 ± 0.89
Triglycerides, mmol/L	1.74 ± 0.82	1.69 ± 0.75	1.71 ± 0.78
Lp(a), g/L	0.559 ± 0.663	0.373 ± 0.385	0.462 ± 0.543
Homocysteine	12.42 ± 3.85	12.22 ± 3.50	12.32 ± 3.66
HbA1c, %	7.4 ± 1.9	7.4 ± 2.0	7.4 ± 1.9
Haematologic and Coagulation Parameters			
Haemoglobin, g/L	119.8 ± 23.7	118.9 ± 24.1	119.4 ± 23.8
Platelet count, ×10^9^/L	255.3 ± 86.9	245.0 ± 102.5	250.1 ± 94.9
Fibrinogen, g/L	4.22 ± 1.29	4.45 ± 1.34	4.34 ± 1.31
INR	1.1 ± 0.1	1.1 ± 0.2	1.1 ± 0.2
APTT, seconds	37.1 ± 31.4	33.7 ± 12.7	35.3 ± 23.5
Pre-Procedural Antithrombotic Therapy			
No antiplatelet therapy	13 (20%)	9 (14%)	22 (17%)
Single antiplatelet therapy	27 (42%)	39 (59%)	66 (51%)
Dual antiplatelet therapy	24 (38%)	18 (27%)	42 (32%)
No anticoagulation	53 (83%)	54 (82%)	107 (82%)
Warfarin	2 (3%)	1 (2%)	3 (2%)
DOAC	9 (14%)	11 (17%)	20 (15%)

Values are presented as means ± SDs for continuous variables and *n* (%) for categorical variables. Percentages are calculated using column totals and may not total 100 due to rounding. Continuous variables were compared using Student’s *t*-test or the Wilcoxon rank-sum test, as appropriate, while categorical variables were compared using the chi-square test or Fisher’s exact test. No adjustments were made for multiple comparisons of baseline characteristics. CABG, coronary artery bypass grafting; COPD, chronic obstructive pulmonary disease; INR, international normalised ratio; APTT, activated partial thromboplastin time; LDL, low-density lipoprotein; Lp(a), lipoprotein(a); DOAC, direct oral anticoagulant.

**Table 2:** Procedural characteristics according to treatment group.

Characteristic	VCD (*n* = 64)	FemoStop™ II Gold (*n* = 66)	Difference (%, 95% CI) †	Total (*n* = 130)
Procedure Type				
Diagnostic only	15 (23.4%)	13 (19.7%)	−3.7 (−18.5 to 12.0)	28 (21.5%)
Interventional procedure	49 (76.6%)	53 (80.3%)	3.7 (−12.0 to 18.5)	102 (78.5%)
Interventional Modality				
Plain balloon angioplasty (POBA)	21 (32.8%)	16 (24.2%)	−8.6 (−25.8 to 9.7)	37 (28.5%)
Drug-coated balloon (DCB)	6 (9.4%)	6 (9.1%)	−0.3 (−11.9 to 11.3)	12 (9.2%)
Covered stent	3 (4.7%)	5 (7.6%)	2.9 (−8.5 to 14.2)	8 (6.2%)
Drug-eluting stent (DES)	2 (3.1%)	2 (3.0%)	−0.1 (−8.4 to 8.2)	4 (3.1%)
Bare-metal stent (BMS)	13 (20.3%)	9 (13.6%)	−6.7 (−22.2 to 9.5)	22 (16.9%)
Atherectomy + DCB	4 (6.3%)	8 (12.1%)	5.8 (−7.3 to 18.5)	12 (9.2%)
Sheath Size (Fr)				
4 Fr	5 (7.8%)	4 (6.1%)	−1.7 (−10.9 to 8.3)	9 (6.9%)
5 Fr	21 (32.8%)	24 (36.4%)	3.6 (−14.4 to 21.2)	45 (34.6%)
6 Fr	29 (45.3%)	34 (51.5%)	6.2 (−12.6 to 23.8)	63 (48.5%)
7 Fr	9 (14.1%)	4 (6.1%)	−8.0 (−20.5 to 4.9)	13 (10.0%)
Haemodynamic Parameters				
Systolic BP, mmHg	142 ± 23	131 ± 18	−11 (−18 to −4) ‡	136 ± 21
Diastolic BP, mmHg	74 ± 12	69 ± 11	−5 (−9 to −1) ‡	71 ± 12
Periprocedural Anticoagulation				
Heparin dose, IU	5480 ± 1120	5390 ± 1050	−90 (−480 to 300) ‡	5434 ± 1085

Values are reported as means ± SDs or *n* (%). Differences for categorical variables are represented as absolute risk differences calculated using the Newcombe–Wilson score method without continuity correction, and those for continuous variables are reported as mean differences with 95% confidence intervals derived from two-sample t statistics. † Difference between FemoStop™ II Gold and VCD. ‡ Mean difference with 95% CI. BP, blood pressure; DCB, drug-coated balloon; DES, drug-eluting stent; BMS, bare-metal stent; Fr, French; IU, international units; POBA, plain old balloon angioplasty; VCD, vascular closure device.

**Table 3:** Efficacy and safety endpoints.

Endpoint	FemoStop™ II Gold	VCD	Difference (95% CI) *	*p*-Value †
	No./Total No. (%)	No./Total No. (%)		
Primary endpoint ‡ (post-procedure)				
Composite groin-site complication (minor or major)	23/66 (34.85)	16/64 (25.00)	9.85 (−6.08 to 25.68)	0.254
Mutually Exclusive Components (Major Takes Precedence)				
Major complication (post-procedure)	4/66 (6.06)	6/64 (9.38)	−3.32 (−16.62 to 10.23)	0.528
Minor complication (post-procedure; minor only)	19/66 (28.79)	10/64 (15.63)	13.16 (−7.17 to 31.93)	0.092
Safety Endpoints—At Discharge				
Major complication (at discharge)	1/66 (1.52)	1/64 (1.56)	−0.05 (−6.93 to 6.66)	>0.999
-Open surgical repair	1/66 (1.52)	1/64 (1.56)	−0.05 (−6.93 to 6.66)	>0.999
-Ultrasound-guided thrombin injection	0/66 (0.00)	0/64 (0.00)	-	-
-Major without invasive intervention	0/66 (0.00)	0/64 (0.00)	-	-
Secondary Endpoint § (30 Days)				
Composite groin-site complication (minor or major)	17/66 (25.76)	12/64 (18.75)	7.01 (−13.25 to 26.39)	0.402
Mutually Exclusive Components (Major Takes Precedence)				
Major complication at 30 days	4/66 (6.06)	3/64 (4.69)	1.37 (−7.63 to 10.43)	0.720
Minor complication at 30 days (minor only; no major)	13/66 (19.70)	9/64 (14.06)	5.64 (−11.33 to 21.85)	0.456
Safety Endpoints—Over 30 Days ¶				
Major complication (total)	4/66 (6.06)	3/64 (4.69)	1.37 (−7.63 to 10.43)	0.720
-Open surgical repair	1/66 (1.52)	1/64 (1.56)	−0.05 (−6.93 to 6.66)	>0.999
-Ultrasound-guided thrombin injection	0/66 (0.00)	2/64 (3.13)	−3.13 (−10.70 to 2.82)	0.240
-Major complication without invasive intervention ††	3/66 (4.55)	0/64 (0.00)	4.55 (−1.54 to 10.78)	0.119

* Differences in absolute risk are expressed as percentage points (FemoStop™ II Gold minus VCD), with 95% confidence intervals calculated using the Newcombe hybrid score method without continuity correction. †: *p*-values are determined using two-sided Fisher’s exact tests. ‡: primary endpoint—composite groin-site complication occurring immediately post-procedure. §: secondary endpoint—composite groin-site complication assessed at 30 days. ¶: safety endpoints represent adjudicated events occurring over 30 days. ††: defined as a major groin-site complication not requiring open surgical repair or ultrasound-guided thrombin injection. Major and minor categories are mutually exclusive at each timepoint; minor denotes minor complications that occurred in the absence of a major complication.

**Table 4:** Modified Poisson risk ratios for groin-site complications.

Timepoint	Outcome	Unadjusted RR † (95% CI)	*p*-Value	Adjusted RR ‡ (95% CI)	Adj. *p*-Value
Post-procedure	Major complication	0.65 (0.20–2.09)	0.480	0.72 (0.20–2.57)	0.620
	Minor complication	1.33 (0.77–2.29)	0.320	1.48 (0.83–2.63)	0.180
	Minor only (no major)	1.84 (0.90–3.78)	0.094	1.91 (0.93–3.92)	0.080
Recovery	Major complication	1.45 (0.45–4.64)	0.530	1.38 (0.42–4.55)	0.600
	Minor complication	1.41 (0.73–2.71)	0.310	1.29 (0.66–2.52)	0.450
Discharge	Major complication	0.97 (0.06–15.30)	0.980	Not estimable §	-
	Minor complication	1.16 (0.38–3.56)	0.790	1.09 (0.34–3.48)	0.880
30 days (Secondary endpoint)	Composite (major or minor)	1.37 (0.71–2.64)	0.360	1.23 (0.63–2.40)	0.540
	Major complication	1.29 (0.31–5.36)	0.720	1.27 (0.30–5.37)	0.750
	Minor only (no major)	1.40 (0.63–3.11)	0.410	1.32 (0.58–2.99)	0.500

†: Unadjusted risk ratios estimated using modified Poisson regression with robust variance. ‡: adjusted risk ratios estimated using modified Poisson regression with robust variance while adjusting for sex, systolic blood pressure prior to sheath removal, and sheath size. §: adjusted estimate not calculable due to sparse event counts and model instability. All models use the VCD as the reference group. Risk ratios > 1 indicate a higher outcome with FemoStop™ II Gold relative to the VCD, and risk ratios < 1 indicate a lower risk.

**Table 5:** Participant-reported pain severity or discomfort after femoral artery access.

Pain Score Category	Post-Procedure		Recovery		Discharge	
	FemoStop™ (*n* = 66)	VCD (*n* = 64)	FemoStop™ (*n* = 66)	VCD (*n* = 64)	FemoStop™ (*n* = 66)	VCD (*n* = 64)
0 (No Pain)	51 (77.3)	36 (58.0)	49 (75.8)	36 (58.6)	59 (89.4)	54 (87.1)
1–3 (Mild)	12 (18.2)	20 (31.3)	13 (19.7)	20 (31.3)	5 (7.6)	5 (7.8)
4–6 (Moderate)	1 (1.5)	5 (7.8)	1 (1.5)	5 (7.8)	2 (3.0)	2 (3.1)
7–10 (Severe)	2 (3.0)	1 (1.6)	2 (3.0)	1 (1.6)	0	0
Global Ordinal Test †	*p* = 0.130		*p* = 0.310		*p* = 0.920	
Effect Size (Cramér’s V)	0.23		0.14		0.03	
Unadjusted OR ‡ (95% CI)	0.42 (0.18–0.97)		0.64 (0.30–1.36)		1.01 (0.32–3.15)	
Adjusted OR § (95% CI)	0.49 (0.22–1.10)		0.60 (0.27–1.33)		0.97 (0.30–3.10)	

†: Two-sided *p*-values derived from Fisher’s exact test comparing the full 4-level ordinal distribution between groups. Cramér’s V quantifies the strength of association between the treatment group and ordinal pain category (range 0–1). ‡: unadjusted proportional-odds logistic regression (VCD reference). §: adjusted proportional-odds logistic regression controlling for sex, systolic blood pressure prior to sheath removal, and sheath size. The proportional-odds assumption was tested and not violated.

## Data Availability

De-identified individual participant data underlying the results reported in this article, together with the data dictionary, are available from the corresponding author upon reasonable request and subject to an appropriate methodologically sound proposal. Statistical code and additional study materials may also be made available upon reasonable request where appropriate.

## Supplementary Materials

The following supporting information can be downloaded at: https://www.mdpi.com/article/10.3390/jcm15114197/s1, Table S1. CONSORT 2025 Checklist for reporting the present randomised controlled trial. Table S2. Groin-site complications at prespecified timepoints. Table S3. Inferential analysis of groin-site complications by treatment group. Table S4. Absolute risk of any pain (Verbal Numerical Rating Scale Score ≥ 1). Table S5. Tests for effect modification in modified Poisson models of groin-site complications. Table S6. Distribution of vascular closure devices and post-procedural complications in the VCD group.
