# Supplementary material for: Mechanical Compression Versus Vascular Closure Devices for Femoral Artery Haemostasis After Peripheral Endovascular Procedures: A Randomised Controlled Trial
Source: J Clin Med. 2026 May 29;15(11):4197. doi: 10.3390/jcm15114197 (PMC13258168; doi:10.3390/jcm15114197)
Supplement: Supplementary file 1 [file jcm-15-04197-s001.zip › jcm-4304005-supplementary.pdf]

SUPPLEMENTARY TABLES

Table S1. CONSORT 2025 Checklist for reporting the present randomised controlled trial.

Section/Topic	No.	CONSORT 2025 Checklist Item Description	Reported in Manuscript
Title and abstract	1a	Identification as a randomised trial	p. 1, Title
Title and abstract	1b	Structured summary of the trial design, methods, results, and conclusions	pp. 2–3, Abstract
Open science	2	Name of trial registry, identifying number (with URL) and date of registration	p. 4, Section 2.1
Open science	3	Where the trial protocol and statistical analysis plan can be accessed	p. 4, Section 2.1
Open science	4	Where and how the individual de-identified participant data, including the data dictionary, statistical code, and any other materials can be accessed	End matter, Data Availability Statement
Funding and conflicts of interest	5a	Sources of funding and other support, and role of funders in the design, conduct, analysis, and reporting of the trial	End matter, Funding
Funding and conflicts of interest	5b	Financial and other conflicts of interest of the manuscript authors	End matter, Conflicts of Interest
Introduction	6	Scientific background and rationale	Introduction
Introduction	7	Specific objectives related to benefits and harms	Introduction
Methods	8	Details of patient or public involvement in the design, conduct, and reporting of the trial	Section 2.1
Methods	9	Description of trial design including type of trial, allocation ratio, and framework	Section 2.1
Methods	10	Important changes to the trial after it commenced including any outcomes or analyses that were not prespecified, with reason	Section 2.1
Methods	11	Settings and locations where the trial was conducted	Section 2.2
Methods	12a	Eligibility criteria for participants	Section 2.2
Methods	12b	Eligibility criteria for sites and for individuals delivering the interventions, if applicable	Section 2.2
Methods	13	Intervention and comparator with sufficient details to allow replication	Section 2.3; Figure 1
Methods	14	Prespecified primary and secondary outcomes, including measurement variable, analysis metric, method of aggregation, and timepoint for each outcome	Section 2.4
Methods	15	How harms were defined and assessed	Section 2.4; Results; Table 3
Methods	16a	How sample size was determined, including all assumptions supporting the sample size calculation	Section 2.5
Methods	16b	Explanation of any interim analyses and stopping guidelines	Section 2.5
Randomisation	17a	Who generated the random allocation sequence and the method used	Section 2.3
Randomisation	17b	Type of randomisation and details of any restriction	Section 2.3
Randomisation	18	Mechanism used to implement the random allocation sequence, describing any steps to conceal the sequence until interventions were assigned	Section 2.3
Randomisation	19	Whether the personnel who enrolled and those who assigned participants to the interventions had access to the random allocation sequence	Section 2.3
Blinding	20a	Who was blinded after assignment to interventions	Section 2.3
Blinding	20b	If blinded, how blinding was achieved and description of the similarity of interventions	Not applicable; open-label trial
Statistical methods	21a	Statistical methods used to compare groups for primary and secondary outcomes, including harms	Section 2.5
Statistical methods	21b	Definition of who is included in each analysis, and in which group	Section 2.5
Statistical methods	21c	How missing data were handled in the analysis	Section 2.5; Figure 2
Statistical methods	21d	Methods for any additional analyses, distinguishing prespecified from post hoc	Section 2.5
Results	22a	For each group, the numbers of participants who were randomly assigned, received intended intervention, and were analysed for the primary outcome	Results; Figure 2
Results	22b	For each group, losses and exclusions after randomisation, together with reasons	Results; Figure 2
Recruitment	23a	Dates defining the periods of recruitment and follow-up for outcomes of benefits and harms	Results
Recruitment	23b	If relevant, why the trial ended or was stopped	Results
Intervention and comparator delivery	24a	Intervention and comparator as they were actually administered	Section 2.3; Figure 1
Intervention and comparator delivery	24b	Concomitant care received during the trial for each group	Section 2.2
Baseline data	25	A table showing baseline demographic and clinical characteristics for each group	Table 1
Numbers analysed, outcomes and estimation	26	For each primary and secondary outcome, by group: number included in the analysis; number with available data; result for each group; effect size and precision; and for binary outcomes, absolute and relative effect size	Tables 3–5; Supplementary Tables S2–S5

Harms	27	All harms or unintended events in each group	Table 3; Supplementary Tables S2–S4
Ancillary analyses	28	Any other analyses performed, including subgroup and sensitivity analyses, distinguishing prespecified from post hoc	Table 4; Supplementary Table S5
Discussion	29	Interpretation consistent with results, balancing benefits and harms, and considering other relevant evidence	Discussion; Conclusions
Discussion	30	Trial limitations, addressing sources of potential bias, imprecision, generalisability, and, if relevant, multiplicity of analyses	Discussion

Table S2. Groin-site complications at prespecified timepoints.

Endpoint	FemoStop™ II Gold (n = 66)	VCD (*n* = 64)	Risk Difference, % (95% CI)	*p* Value	Adjusted RR (95% CI)
**Post-procedure**					
**Composite (minor or major)**	23/66 (34.8)	16/64 (25.0)	9.8 (−6.0 to 25.7)	0.220	1.39 (0.83–2.33)
**Major complication**	4/66 (6.1)	6/64 (9.4)	−3.3 (−12.4 to 5.8)	0.480	0.69 (0.20–2.32)
**Minor complication**	22/66 (33.3)	16/64 (25.0)	8.3 (−8.3 to 24.9)	0.320	1.34 (0.78–2.29)
**Recovery**					
**Composite (minor or major)**	17/66 (25.8)	12/64 (18.8)	7.0 (−7.8 to 21.8)	0.340	1.30 (0.67–2.52)
**Major complication**	6/66 (9.1)	4/64 (6.3)	2.8 (−6.8 to 12.4)	0.750	1.44 (0.42–4.93)
**Minor complication**	16/66 (24.2)	11/64 (17.2)	7.0 (−7.5 to 21.5)	0.360	1.35 (0.68–2.69)
**At hospital discharge**					
**Composite (minor or major)**	7/66 (10.6)	6/64 (9.4)	1.2 (−9.4 to 11.8)	0.810	1.10 (0.39–3.11)
**Major complication**	1/66 (1.5)	1/64 (1.6)	−0.1 (−4.5 to 4.3)	>0.990	0.98 (0.06–15.4)
**Minor complication**	6/66 (9.1)	5/64 (7.8)	1.3 (−8.9 to 11.5)	0.750	1.16 (0.37–3.66)
**At 30 days**					
**Composite (minor or major)**	17/66 (25.8)	12/64 (18.8)	7.0 (−13.3 to 26.4)	0.360	1.28 (0.66–2.49)
**Major complication**	4/66 (6.1)	3/64 (4.7)	1.4 (−6.9 to 9.7)	0.720	1.27 (0.30–5.37)
**Minor complication**	13/66 (19.7)	9/64 (14.1)	5.6 (−11.3 to 21.8)	0.460	1.34 (0.63–2.86)

Risk differences were calculated as FemoStop minus VCD using the Newcombe method, without continuity correction, for independent proportions. *p* values are two-sided and were calculated using Fisher's exact test. Adjusted risk ratios were estimated using modified Poisson regression with robust variance, adjusted for sex, systolic blood pressure prior to sheath removal, and sheath size (Fr). VCD was the reference group. Composite outcomes include major and minor complications.

Table S3. Inferential analysis of groin-site complications by treatment group.

Outcome	FemoStop™ II Gold (*n* = 66)	VCD (*n* = 64)	Relative Risk (95% CI)	Adjusted RR (95% CI)	Risk Difference % (95% CI)	*p* Value
**Post-procedure (Primary Endpoint)**						
Composite (minor + major)	23 (34.8%)	16 (25.0%)	1.39 (0.83–2.33)	1.28 (0.74–2.21)	+9.8 (−6.1 to 25.6)	0.22
Major	4 (6.1%)	6 (9.4%)	0.65 (0.19–2.18)	0.72 (0.20–2.57)	−3.3 (−12.7 to 6.1)	0.53
Minor	22 (33.3%)	16 (25.0%)	1.33 (0.78–2.27)	1.22 (0.70–2.14)	+8.3 (−8.7 to 24.9)	0.32
**Recovery**						
Composite	17 (25.8%)	12 (18.8%)	1.37 (0.71–2.65)	1.29 (0.64–2.58)	+7.0 (−7.9 to 21.8)	0.34
Major	6 (9.1%)	4 (6.3%)	1.45 (0.42–5.02)	1.31 (0.36–4.78)	+2.8 (−7.1 to 12.5)	0.75
Minor	16 (24.2%)	11 (17.2%)	1.41 (0.70–2.83)	1.32 (0.63–2.78)	+7.0 (−8.3 to 21.6)	0.36
**Discharge**						
Composite	7 (10.6%)	6 (9.4%)	1.13 (0.39–3.26)	1.08 (0.36–3.22)	+1.2 (−9.8 to 12.1)	0.81
Major	1 (1.5%)	1 (1.6%)	0.97 (0.06–15.4)	0.95 (0.06–14.8)	−0.1 (−4.6 to 4.5)	1.00
Minor	6 (9.1%)	5 (7.8%)	1.17 (0.38–3.62)	1.11 (0.35–3.51)	+1.3 (−9.3 to 11.7)	0.75
**30-Day Follow-Up (Secondary Endpoint)**						
Composite (minor + major)	17 (25.8%)	12 (18.8%)	1.37 (0.71–2.64)	1.23 (0.63–2.40)	+7.0 (−13.3 to 26.4)	0.40

Risk differences are expressed as percentage points, FemoStop™ II Gold minus VCD, with 95% confidence intervals calculated using the Newcombe hybrid score method without continuity correction. *p* values are two-sided Fisher's exact tests. Adjusted risk ratios were estimated using modified Poisson regression with robust variance, adjusted for sex, systolic blood pressure prior to sheath removal, and sheath size. Major and minor categories are not mutually exclusive unless specified as "minor only."

Table S4. Absolute risk of any pain (Verbal Numerical Rating Scale Score ≥ 1).

Timepoint	FemoStop™ II Gold (*n* = 66)	VCD (*n* = 64)	Risk Difference % (95% CI)	*p* Value	Unadjusted RR (95% CI)	*p* Value	Adjusted RR (95% CI)	Adj. *p* Value
Post-procedure	15/66 (22.7)	26/64 (40.6)	−17.9 (−38.6 to 4.7)	0.038	0.56 (0.33–0.93)	0.026	0.63 (0.38–1.03)	0.066
Recovery	17/66 (25.8)	26/64 (40.6)	−14.9 (−36.1 to 8.0)	0.093	0.64 (0.40–1.03)	0.067	0.71 (0.44–1.14)	0.156
Discharge	7/66 (10.6)	7/64 (10.9)	−0.3 (−15.7 to 14.9)	1.000	0.97 (0.37–2.53)	0.952	0.98 (0.36–2.64)	0.972

Risk differences are expressed as percentage points, FemoStop minus VCD, and were calculated using the Newcombe hybrid score method, Wilson score intervals without continuity correction. Two-sided *p* values were derived from Fisher's exact test comparing proportions of participants reporting any pain, VNRS ≥ 1, versus no pain, VNRS = 0. Unadjusted risk ratios were estimated using modified Poisson regression with robust standard errors. Adjusted risk ratios were estimated using modified Poisson regression with robust standard errors, adjusted for sex, systolic blood pressure prior to sheath removal, and sheath size. VCD served as the reference group.

Table S5. Tests for effect modification in modified Poisson models of groin-site complications.

Interaction Tested	Outcome	Timepoints Included	Interaction Term	Wald χ^2 (df)	*p* Value
Treatment × Time	Composite groin-site complication (minor or major)	Post-procedure, recovery, discharge	Treatment × Time (3-level)	0.56 (2)	0.753
Treatment × Sheath Size	Composite complication (post-procedure)	Post-procedure	Treatment × sheath size (categorical, 4–7 Fr)	14.8 (3)	<0.001
Treatment × Sheath Size	Composite complication (30 days)	30-day follow-up	Treatment × sheath size (categorical, 4–7 Fr)	15.2 (3)	<0.001

Modified Poisson regression with log link and robust standard errors was used to estimate relative risks. Interaction terms were evaluated using Wald tests of joint significance. The treatment × time interaction assessed whether the relative risk associated with FemoStop™ II Gold versus VCD differed across in-hospital timepoints, post-procedure, recovery, and discharge. The treatment × sheath size interaction assessed whether the association between treatment allocation and complication risk varied by sheath size category, 4–7 Fr. All models used VCD as the reference group and were estimated using modified Poisson regression with robust variance. All *p* values were two-sided.

Table S6. Distribution of Vascular Closure Devices and Post-Procedural Complications in the VCD Group

Vascular closure device	Mechanism/description	Total VCD use, n (%)	Post-procedural complications, n/N (%)	Exact 95% CI
Angio-Seal™	Collagen-based vascular closure device	3 (4.7)	1/3 (33.3)	0.8–90.6
MynxGrip™	Polyethylene glycol sealant-mediated closure device	22 (34.4)	5/22 (22.7)	7.8–45.4
FemoSeal™	Collagen-based/clip-mediated vascular closure device	24 (37.5)	6/24 (25.0)	9.8–46.7
Perclose ProGlide™	Suture-mediated vascular closure device	15 (23.4)	4/15 (26.7)	7.8–55.1
Total VCD group analysed		**64 (100.0)**	**16/64 (25.0)**	**15.0–37.4**

Exploratory statistical comparison: Fisher's exact test across VCD subtypes, $p = 0.94$; Cramér's V = 0.07. Values are presented as n (%) or n/N (%). Percentages for total vascular closure device (VCD) use were calculated using the total analysed VCD cohort as the denominator (n = 64). Percentages for post-procedural complications were calculated using the number of participants receiving each individual VCD platform as the denominator. Exact 95% confidence intervals were calculated using the Clopper–Pearson method because of the small subgroup sample sizes and low event counts. CI, confidence interval; VCD, vascular closure device.
